# Evaluation of a flavonoid library for inhibition of interaction of HIV-1 integrase with human LEDGF/p75 towards a structure–activity relationship

**DOI:** 10.1080/07853890.2022.2081869

**Published:** 2022-06-06

**Authors:** Zhi-Hui Yin, Hao-Li Yan, Yu Pan, Da-Wei Zhang, Xin Yan

**Affiliations:** a Institute of Bioinformatics and Medical Engineering, School of Electrical and Information Engineering, Jiangsu University of Technology, Changzhou, China; b School of Computer Engineering, Jiangsu University of Technology, Changzhou, China; c First Hospital of Shanxi Medical University, Taiyuan, China; d Center for Food and Drug Evaluation & Inspection of Henan, Zhengzhou, China

**Keywords:** Flavonoid, integrase, integrase-LEDGF interaction, HIV-1

## Abstract

**Background:** Proteinśprotein interaction (PPI) between lens epithelium-derived growth factor (LEDGF/p75) and human immunodeficiency virus (HIV) integrase (IN) becomes an attractive target for anti-HIV drug development.

**Methods:** The blockade of this interaction by small molecules could potentially inhibit HIV-1 replication. In this study, a panel of 99 structurally related flavonoids were was tested, concerning their ability to inhibit IN-LEDGF/p75 interaction, using a homogeneous time time-resolved fluorescence (HTRF) assay.

**Results:** From the obtained results, it was possible to observe that the flavonoid with hydroxyl group in C3-, C4-, C5- and C7-position on the A-ring, C4′- and C5′-position of the B-ring, a carbonyl group of the C-ring, was more active against IN-LEDGF/p75 interaction, through competitive inhibition. Moreover, the binding modes of representative compounds, including myricetin, luteolin, dihydrorobinetin, naringenin, epicatechin, genistein and helichrysetin, were analyzedanalysed by molecular docking. Biolayer interferometry assay confirmed that these representative compounds disrupted the PPI by binding to IN with KD values ranging from 1.0 to 3.6 µM.

**Conclusion:** This study presents the first to quantitative comparation of the effect of flavonoids with different structural subclasses on IN-LEDGF/p75 interaction. Our findings provide new insights into the development of inhibitors targeting IN-LEDGF/p75 interaction using flavonoids.

**Key Messages**
- HIV-1 integrase (IN)-LEDGF/p75 interaction is an attractive target for antiviral drug development.
- For the first time, the structure-activity relationship of flavonoids belonging to seven flavonoidic subclasses on IN-LEDGF/p75 interaction was determined.
- This study comprehends an HTRF-based screening system, biolayer interferometry and an in silico molecular docking analysis.

## 1. Introduction

HIV-1 integrase (IN), a virus-encoded enzyme, plays a vital role in viral replication. IN has no counterparts in mammalian, this is considered an appealing target for anti-HIV-1 drugs [1–3]. IN catalyses the integration of viral DNA into the host genome in a two-step process, 3′-processing and strand transfer [4–6]. The catalytic core domain of IN, critical to its enzymatic function, comprises the acidic triad DDE (D64, D116, E15) motif, which coordinates two magnesium ions [6]. Chelation of the divalent ion can impair the function of IN, providing a good development strategy for IN inhibitors [7]. In fact, three clinically available IN inhibitors (including raltegravir, elvitegravir, and dolutegravir) bind the magnesium ions through the keto-enol carboxyl moiety and form hydrophobic interactions with the DDE motif [8].

Although significant progress has been achieved in developing IN inhibitors, there is a continued need for developing new inhibitors targeting IN due to the emergence of inhibitor-resistant viruses and the nucleotide flexibility of the HIV-1 genome [9,10]. Human lens epithelium-derived growth factor/p75 (LEDGF/p75) binds and stimulates IN activity by tethering IN to host cell chromatins [11]. Blockage of IN-LEDGF/p75 interaction can inhibit HIV-1 viral replication [12]. Hence, IN–LEDGF/p75 interaction represents an attractive and promising target for the development of the novel anti-HIV drug.

Flavonoids are natural products with the 2-phenylchromen-4-one (2-phenyl-1-benzopyran-4-one) structure, which exert versatile biological activities such as antioxidative, anti-inflammatory, antiallergic, antiviral, and anticancer activities, as well as a beneficial effect on cardiovascular diseases [13,14]. Flavonoids, as excellent metal chelators, potently blocked HIV-1 IN activity and displayed effective antiviral activity *in vitro* [15–18]. Flavonoid-based HIV-1 IN inhibitors also disrupted the IN-LEDGF/p75 interaction in cell-based assays [16,19]. However, it is not clear if the structure-activity relationship (SAR) for IN-LEDGF/p75 interaction inhibitory activity from a different subclass of flavonoids varied due to the difference in the number and position of the hydroxy groups around the flavonoid ring system. To fill this gap, a total of 99 flavonoids including flavonols, flavanones, flavanonols, flavones, flavan, chalcones and isoflavones, were investigated for their inhibitory activity against IN-LEDGF/p75 interaction. This study comprehends an HTRF-based screening system, biolayer interferometry and an in silico molecular docking analysis.

## 2. Material and methods

### 2.1. Agents and inhibitor libraries

Anti-6His-XL665 Cryptate antibodies and anti-GST-Eu Cryptate antibodies, white 384-shallow well microplate were purchased from PerkinElmer. 96-well Black microplates were purchased from Greiner Bio-One. Streptavidin (SA) biosensors were purchased from Sartorius ForteBio. EZ-Link™ Sulfo-NHS-LC-LC-Biotin was purchased from Thermo Fisher Scientific. BI224436 was purchased from MedChem Express (Shanghai, China). Compounds were obtained from National Compound Resource Centre (Shanghai, China) and stock solutions (10 mM) were stored protected from light at −80 °C. Ni-NTA resin and GST resin were purchased from Smart-Lifesciences (Changzhou, China). All general biochemical reagents were obtained from AMRESCO (Solon, USA).

### 2.2. Recombinant protein expression and purification

The LEDGF/p75 IBD domain encoding sequence was amplified by PCR from plasmid pGEX-4T-LEDGF/p75 (conserved in our lab) and subcloned into the BamH I-Sal I sites of the vector pGEX-4T-1, creating pGEX-4T-IBD construct for expression of N-terminal GST-tagged IBD. The full HIV IN coding sequence, including soluble double mutant F185K/C280S, was PCR amplified from pET28a-IN (conserved in our lab) and sub-cloned into Nco I-Xho I sites of the vector pET28a to create the pET28a-IN-C for expression of C-terminal His6–tagged IN. IBD, IN were expressed and purified as previously described [20]. The concentrations of all proteins were determined by the Bradford assay with bovine serum albumin (BSA) as a standard. 12% SDS-PAGE was performed to determine the purity of proteins.

### 2.3. Inhibition assays of in-LEDGF/p75 *in vitro*

The inhibitory activities against the IBD-IN interaction were tested using HTRF assays according to a previous study [21]. The assay was performed in a white 384-shallow well microplate. “Protein MIX” was prepared by mixing His_6_-HIV-1 IN (final concentration of 50 nM) and GST-IBD (final concentration of 25) in the assay buffer (25 mM Tris-HCl pH 7.5, 150 mM NaCl, 1 mg/ml BSA, 0.1% NP40, 2 mM MgCl_2_). Eight microliters “protein MIX” was added to each well in a white 384-shallow well microplate. To the 8 μl “protein MIX”, 2 μl of DMSO or compound (50 µM) dissolved in DMSO was added and mixed thoroughly. The concentrations of DMSO in the assay should be no more than 4%. The plate was incubated for 30 min at 25 °C. Then 10 μl premixed antibodies were added and mixed. The assay plate was incubated in the dark for 1 h at 25 °C. DMSO instead of the compound was used as a negative control. BI224436 was used as a positive compound for inhibition of IN-LEDGF/p75 interaction [21]. HTRF measurement wea performed in an Envision 2102 multilabel reader. Data were analysed and visualised in GraphPad Prism 5.0.

### 2.4. Biolayer interferometry assay

A protein binding assay was performed by biolayer interferometry (BLI) as described previously [22]. First, purified recombinant LEDGF/p75 protein was biotinylated using the Thermo EZLink long-chain biotinylation reagent. Then, biolayer interferometry (BLI) assay was performed using an OctetRED96 instrument from PALL/ForteBio. All assays were run at 30 °C with continuous 1000 rpm shaking. PBS with 0.01% Tween-20 was used as the assay buffer. Briefly, Biotinylated LEDGF protein was tethered on Super Streptavidin (SSA) biosensors (ForteBio) by dipping sensors into 200 µl per well 50 µg/ml protein solutions. The measurement processes were all under computer control. Program procedures were established as follows: for the initial step, biosensors were washed in assay buffer to form a baseline; the biosensors labelled with biotin-LEDGF were exposed to diluted a series of three, twofold diluted compounds for the association, and then the biosensors were moved back into assay buffer to disassociate. Data were fit globally and generated automatically by Octet User software (version 9.0.0.10; Fortebio).

### 2.5. Molecular docking

The structural models of integrase catalytic domain and catechins were generated by molecular docking. The receptor structure was extracted from the complex structure of integrase and compound BI 224436 (PDB id: 4NYF). The docking site was set to the BI 224436 binding pocket, which is for LEDGF/p75 IBD domain binding. Then molecular docking was performed by SwissDock [23,24] and the conformation with the highest binding affinity was kept for model representation by PyMOL.

## 3. Results

HIV-1 IN interacts with LEDGF/p75, by which it is tethered to chromatin. Within the interaction interface, residues 168–171 of IN form a hydrogen-bond network with IBD, and a hydrophobic patch with the side-chain residues (ILE365, PHE406, and VAL408) of LEDGF/p75. The residue ASP366 of LEDGF/p75 forms a bidentate hydrogen bond with the IN residues (GLU170 and HIS17) in chain A. Residue ILE365 inserts into a hydrophobic pocket formed by residues (Thr174 and Met178) in chain A as well as residues LEU102, ALA128, ALA129, and TRP132 in chain B [25]. In the present study, quantitative comparations of the effect of flavonoids belonging to 7 different structural subclasses on IN-LEDGF/p75 interaction were determined and discussed. All recombinant proteins were affinity purified from E. coli >90% as assessed by 12% SDS-PAGE (Figure S1). BI224436, as a positive control, showed dose-dependent inhibition of IN-LEDGF/p75 interaction (Figure S2). with an IC_50_ of 18.8 nM which was comparable to the previous experiment result of 11.0 nM [21], validating that the assay was effective and suitable for drug screening applications.

### 3.1. IN-LEDGF/p75 interaction inhibitory activity of flavonols

**Figure 1.**
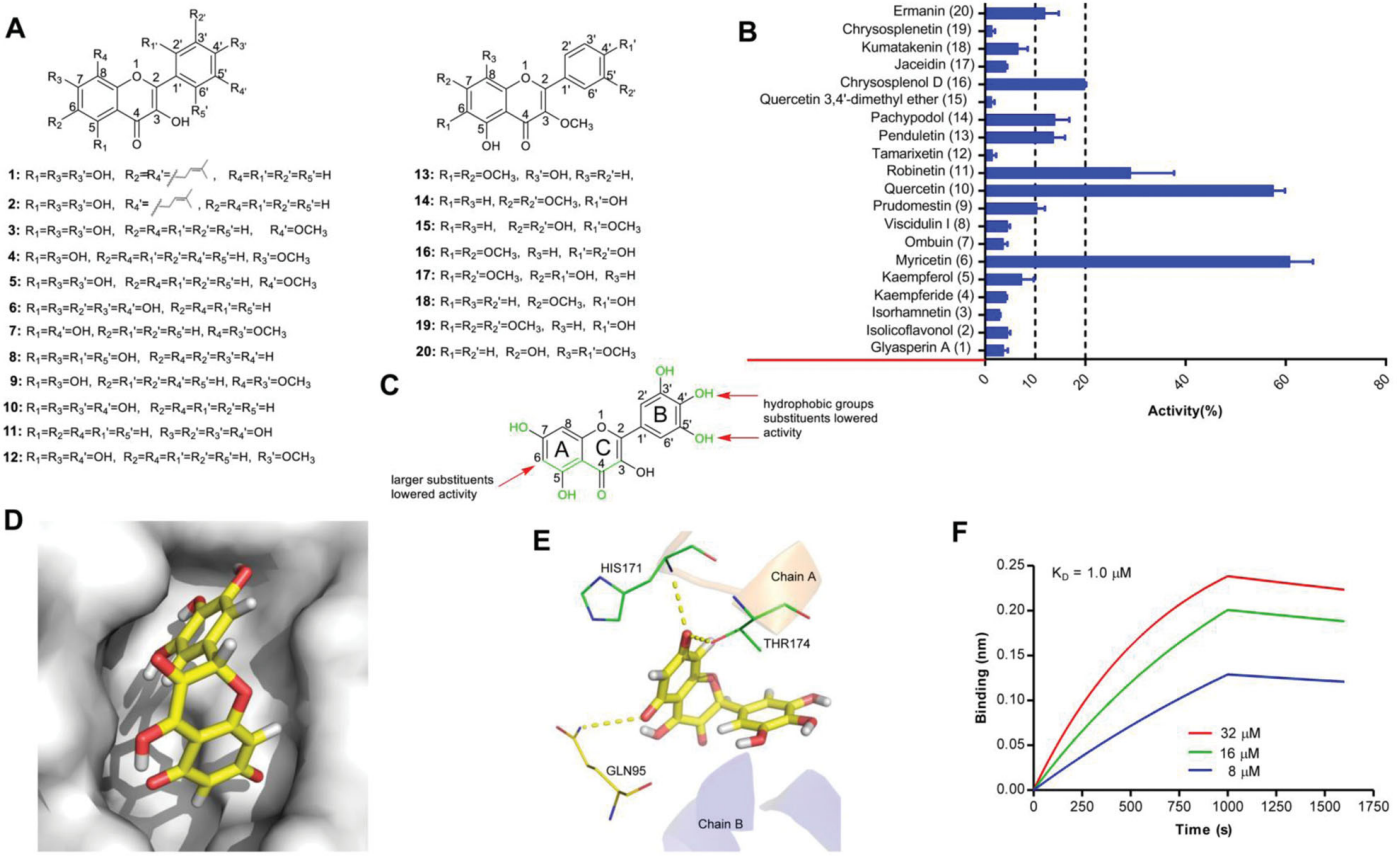
(A) Chemical structures of the studied flavonols. (B) The inhibitory effects of 20 flavonols (50 μM, final concentration) against IN-LEDGF/p75 interaction. (C) Proposed semi-quantitative structure-activity relationships for flavonols. Structural features decreasing the inhibition of IN-LEDGF/p75 interaction are marked in red colour. Structural characteristics increasing the inhibitory potential of flavonols are shown in green. Plausible binding modes of myricetin *via* Chimaera. The binding pocket of the IN CCD was illustrated as (D) surface and (E) thin lines for key residues (PDB accession code 4NYF). Myricetin is coloured yellow. Yellow dashed lines represent H-bonding interactions between the ligand and receptor. (F) Sensorgram of the interaction between the immobilised IN and myricetin.

Twenty flavonols (Figure 1A) were tested in biochemical assays for their inhibition of IN–LEDGF/p75 interaction. These compounds showed some inhibitory potential (Figure 1B). Among them, 3 compounds showed pronounced (>20%), 5 compounds lower, but still distinguishable (between 10% and 20%), and 12 compounds weak (less than 10%) inhibitory activity. Compounds that bear both catechol and ketoenol structure (10, 16), effectively inhibited IN–LEDGF/p75 interaction as described in a previous study [16]; in contrast, compounds with only one ketoenol moiety were inactive in inhibiting the interaction (e.g. compounds 5, 8, 9, 13, 18, 20). The ketoenol compounds with a pyrogallol group also efficiently blocked IN–LEDGF/p75 interaction (6); compounds containing one pyrogallol moiety only (11) were also active in disrupting the interaction but was less active than compound with an additional ketoenol structure (6). IN–LEDGF/p75 inhibitory activity was reduced when the catechol structure was replaced by hydrophobic groups (e.g. compounds 1, 2, 3, 4, 7, 12, 14, 15, 17, 19). Based on these observations, a semi-quantitative assumption for the structural determinants influencing the flavonols′ inhibitory activities on IN- LEDGF/p75 interaction was developed (Figure 1C). Plausible binding modes of one representative compound *via* induced-fit docking were shown in Figure 1D and E. The docking poses of myricetin revealed that the 7-hydroxyl group of the A-ring formed hydrogen bonds with the main-chain amides of HIS171 and THR174 in chain A and 5-hydroxyl group of the A-ring formed a hydrogen bond with residue GLN 95 in the chain B. BLI assay confirmed that myricetin bound directly to IN with a K_D_ value of 1.0 µM (Figure 1F).

### 3.2. IN-LEDGF/p75 interaction inhibitory activity of flavones

**Figure 2.**
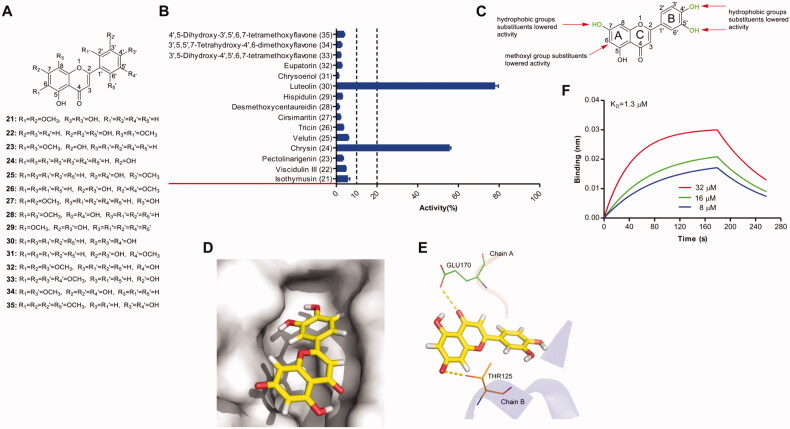
(A) Chemical structures of the studied flavones. (B) The inhibitory effects of 15 flavonols (50 μM, final concentration) against IN-LEDGF/p75 interaction. (C) Proposed semi-quantitative structure-activity relationships for flavones. Structural features decreasing the inhibition of IN-LEDGF/p75 interaction are marked in red colour. Structural characteristics increasing the inhibitory potential of flavones are shown in green. Putative binding modes of luteolin *via* Chimaera. The binding pocket of the IN CCD was illustrated as (D) surface and (E) thin lines for key residues (PDB accession code 4NYF). Luteolin is coloured yellow. Yellow dashed lines represent H-bonding interactions between the Luteolin and receptor. (F) Sensorgram of the interaction between the immobilised IN and luteolin.

Fifteen flavones (Figure 2A) were evaluated on their inhibitory activity against IN–LEDGF/p75 interaction. As shown in Figure 2B, 2 compounds displayed pronounced inhibitory activity, other 13 compounds exhibited weak inhibitory activity. The most potent one is Luteolin (30), which is a ketoenol compound with a catechol group. Regardless of the catechol structure co-occurrence with ketoenol moiety, the introduction of the hydroxy group into the 7-position was beneficial for the IN–LEDGF/p75 inhibition as exemplified by compound 24. IN–LEDGF/p75 inhibitory activity was reduced when the catechol structure or 7-hydroxy was replaced by hydrophobic groups or methoxyl group was introduced into the 6-position. Based on the above data, a semi-quantitative assumption for the structural determinants influencing the flavones′ inhibitory activities on IN-LEDGF/p75 interaction was proposed (Figure 2C). The predicted binding modes of one representative compound luteolin were presented in Figure 2D and E. The molecular docking analysis revealed that the carbonyl group of the C-ring formed hydrogen bonds with the main-chain amides of GLU170 in chain A and the 7-hydroxyl group of A-ring formed a hydrogen bond with residue THR125 in the chain B. BLI assay confirmed the direct binding of luteolin to IN with a K_D_ value of 1.3 µM (Figure 2F).

### 3.3. IN-LEDGF/p75 interaction inhibitory activity of flavanonols

**Figure 3.**
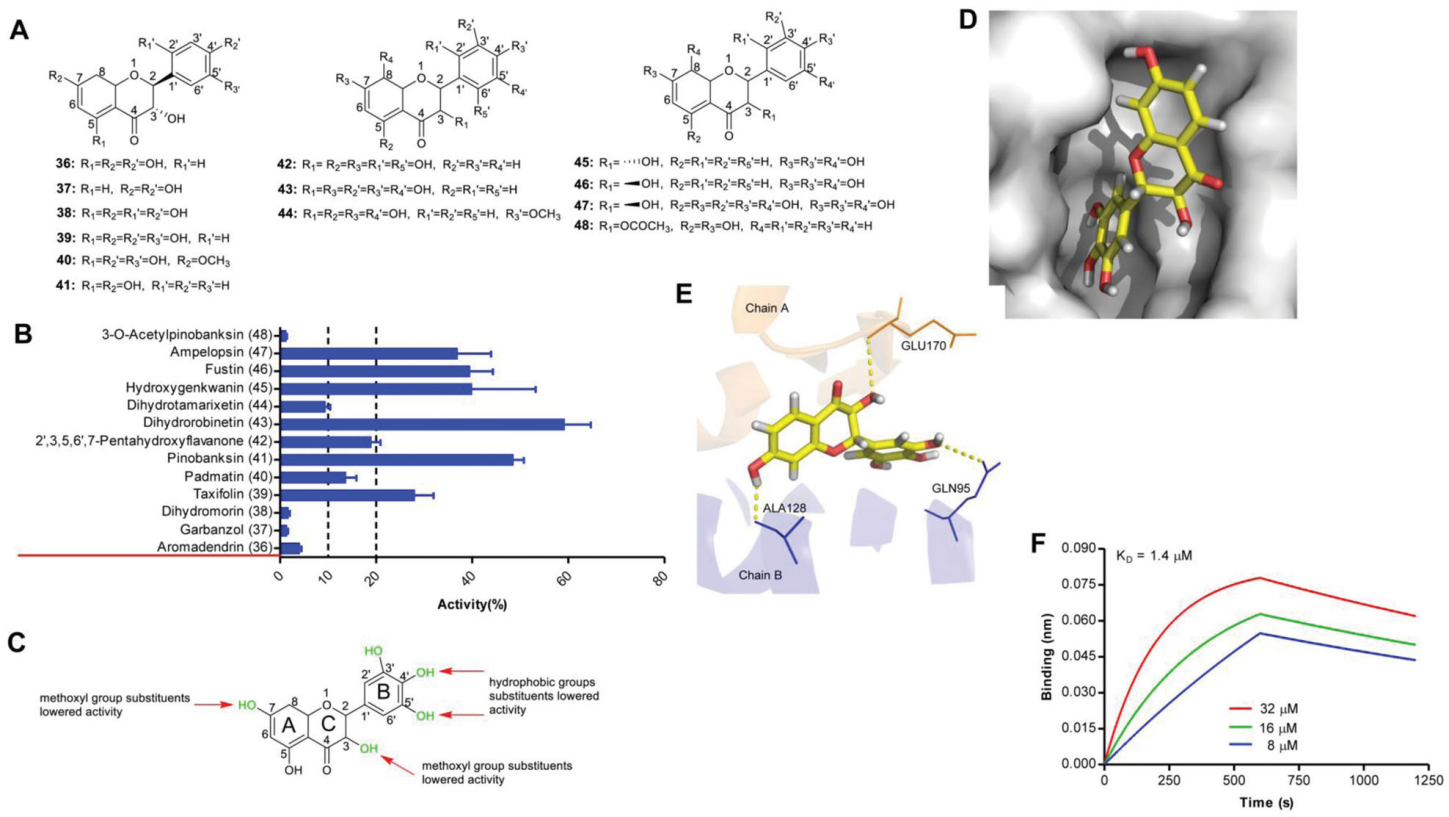
(A) Chemical structures of the studied flavanonols. (B) The inhibitory effects of 13 flavonols (50 μM, final concentration) against IN-LEDGF/p75 interaction. (C) Proposed semi-quantitative structure-activity relationships for flavanonols. Structural features decreasing the inhibition of IN-LEDGF/p75 interaction are marked in red colour. Structural characteristics increasing the inhibitory potential of flavanonols are shown in green. Predicted binding modes of dihydrorobinetin *via* Chimaera. The binding pocket of the IN CCD was illustrated as (D) surface and (E) thin lines for key residues (PDB accession code 4NYF). Dihydrorobinetin is coloured in yellow. Yellow dashed lines represent H-bonding interactions between the dihydrorobinetin and receptor. (F) Sensorgram of the interaction between the immobilised IN and dihydrorobinetin.

Thirteen flavanonols (Figure 3A) were assessed for their ability to block IN–LEDGF/p75 interaction in a HTRF assay. The results indicated that 6 compounds exhibited pronounced (>20%), 3 compounds lower, but still distinguishable (between 10% and 20%), and 4 compounds weak (less than 10%) inhibitory activity (Figure 3B). The two ketoenol compounds (43, 47) with a pyrogallol group efficiently blocked IN–LEDGF/p75 interaction, suggesting that the pyrogallol group was favoured for activity. The 7-hydroxy or 3-hydroxy played an important role in the disruption of IN-LEDGF/p75 interaction essential for inhibiting IN-LEDGF/p75 interaction, and the replacement of 7-hydroxy or 3 hydroxy by methoxyl group led to the drop of the protein-protein interaction disruption (39 vs 40, 41 vs 48). Catechol structure exerted more impact on potency than ketoenol moiety had (36 vs 37, 45 vs 41). Based on these findings, a semi-quantitative assumption was proposed for the structural determinants affecting the IN-LEDGF/p75 interaction by flavanonols (Figure 3C). The predicted binding modes of one representative compound dihydrorobinetin were shown in Figure 3D and E. It was observed that the 3-hydroxyl group of the C-ring formed hydrogen bonds with the main-chain amides of GLU170 in chain A, the 7-hydroxyl group of A-ring formed a hydrogen bond with residue ALA128 in the chain B and 4′-hydroxyl group of B-ring formed a hydrogen bond with residue GLN95. Moreover, dihydrorobinetin directly interacted with IN with a K_D_ value of 1.4 µM (Figure 3F).

### 3.4. IN-LEDGF/p75 interaction inhibitory activity of flavanones

**Figure 4.**
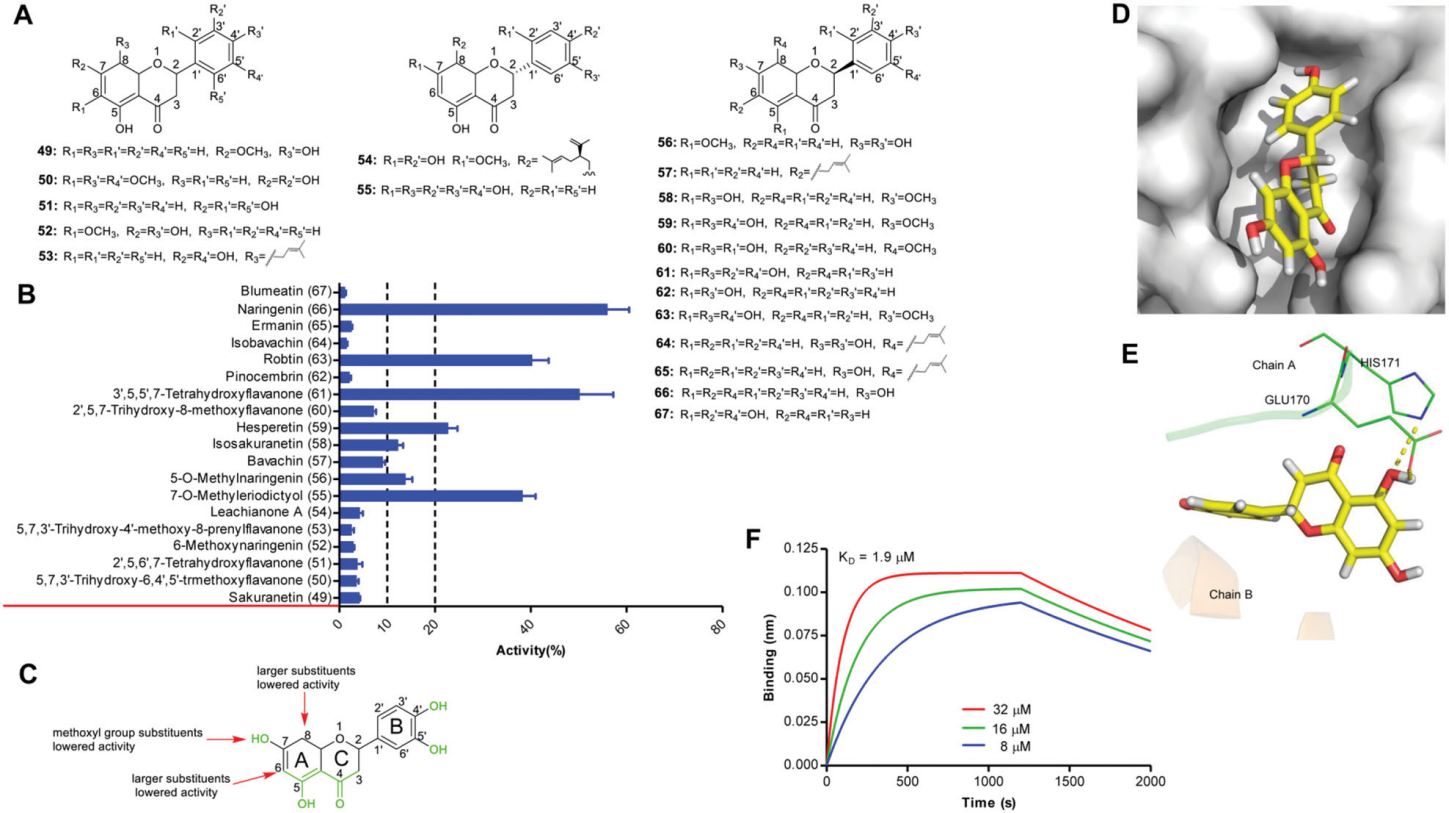
(A) Chemical structures of the studied flavanones. (B) The inhibitory effects of 13 flavanones (50 μM, final concentration) against IN-LEDGF/p75 interaction. (C) Proposed semi-quantitative structure-activity relationships for flavanones. Structural features decreasing the inhibition of IN-LEDGF/p75 interaction are marked in red colour. Structural characteristics increasing the inhibitory potential of flavanones are shown in green. Putative binding modes of naringenin *via* Chimaera. The binding pocket of the IN CCD was illustrated as (D) surface and (E) thin lines for key residues (PDB accession code 4NYF). Naringenin is coloured in yellow. Yellow dashed lines represent H-bonding interactions between the naringenin and receptor. (F) Sensorgram of the interaction between the immobilised IN and naringenin.

An HTRF-based assay was used to assess the inhibitory activity of 13 flavanones against IN–LEDGF/p75 interaction (Figure 4A). The results showed that 7 compounds displayed pronounced inhibitory activity against IN–LEDGF/p75 interaction (Figure 4B). The replacement of 7-hydroxy by methoxyl group, or the larger 6-substituents or 8-substituents caused a loss of activity the protein-protein interaction disruption (e.g. compounds 49, 50, 52, 53, 54, 57, 60, 64, 65, 67). For these compounds, ketoenol group or catechol group still played an important role in the disruption of protein-protein interaction (56 vs 66, 67 vs 61). Proposed semi-quantitative structure-activity relationships for flavanones′ inhibitory activities on IN-LEDGF/p75 interaction were shown in Figure 4C. The binding mode analysis (Figure 4D and E) of one representative compound naringenin revealed that the 5-hydroxyl group of the A-ring formed hydrogen bonds with the main-chain amides of GLU170 and HIS 171 in chain A. The direct binding of naringenin to IN with a K_D_ value of 1.4 µM was confirmed by BLI assay (Figure 4F).

### 3.5. IN-LEDGF/p75 interaction inhibitory activity of flavans

**Figure 5.**
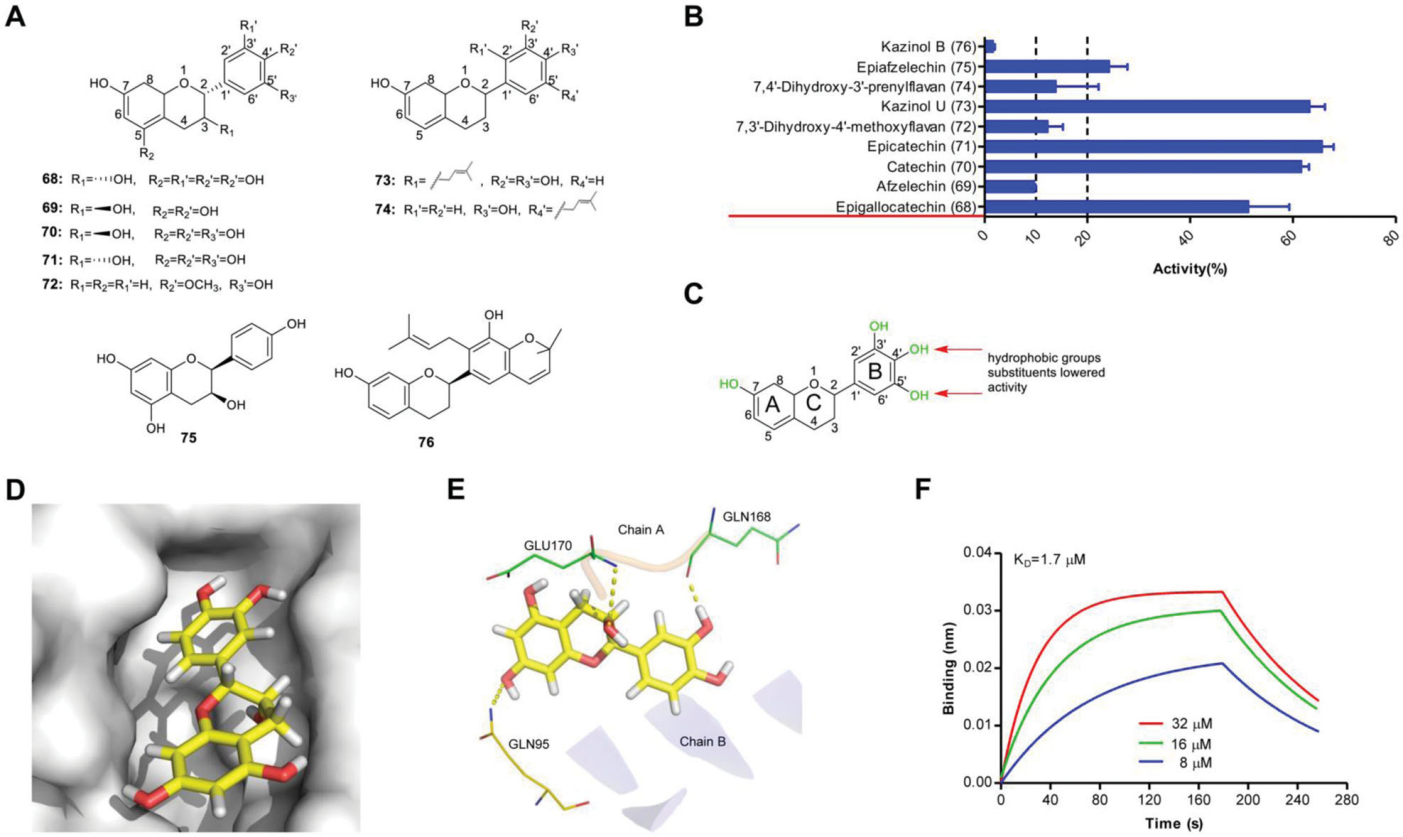
(A) Chemical structures of the studied flavan. (B) The inhibitory effects of 9 flavan (50 μM, final concentration) against IN-LEDGF/p75 interaction. (C) Proposed semi-quantitative structure-activity relationships for flavan. Structural features decreasing the inhibition of IN-LEDGF/p75 interaction are marked in red colour. Structural characteristics increasing the inhibitory potential of flavan are shown in green. Plausible binding modes of naringenin *via* Chimaera. The binding pocket of the IN CCD was illustrated as (D) surface and (E) thin lines for key residues (PDB accession code 4NYF). Epicatechin is coloured yellow. Yellow dashed lines represent H-bonding interactions between the epicatechin and receptor. (F) Sensorgram of the interaction between the immobilised IN and epicatechin.

Nine flavans (Figure 5A) were tested in biochemical assays for their ability to disrupt IN–LEDGF/p75 interaction. As shown in Figure 5B, among the tested compounds, 5 compounds showed more potent inhibitory activity against IN–LEDGF/p75 interaction. Pyrogallol group or catechol-containing compounds effectively blocked IN–LEDGF/p75 interaction (e.g. compounds 68, 70, 71). None of these compounds contained ketoenol moiety, suggesting that this group exerts little impact on the potency of flavan. Accordingly, structural characteristics influencing the flavans′ inhibitory activities on IN-LEDGF/p75 interaction were depicted in Figure 5C. The putative binding modes of one representative compound epicatechin showed that the 3-hydroxyl group of the C-ring formed hydrogen bonds with the main-chain amides of GLU170, 4′-hydroxyl group of the B-ring formed hydrogen bonds with GLN168 in chain A, and 7-hydroxyl group of A-ring formed a hydrogen bond with residue GLN95 in the chain B (Figure 5D and E). BLI assay confirmed that epicatechin exhibited a relatively strong binding affinity to IN with a K_D_ value of 1.7 µM (Figure 5F).

### 3.6. IN-LEDGF/p75 interaction inhibitory activity of isoflavones

**Figure 6.**
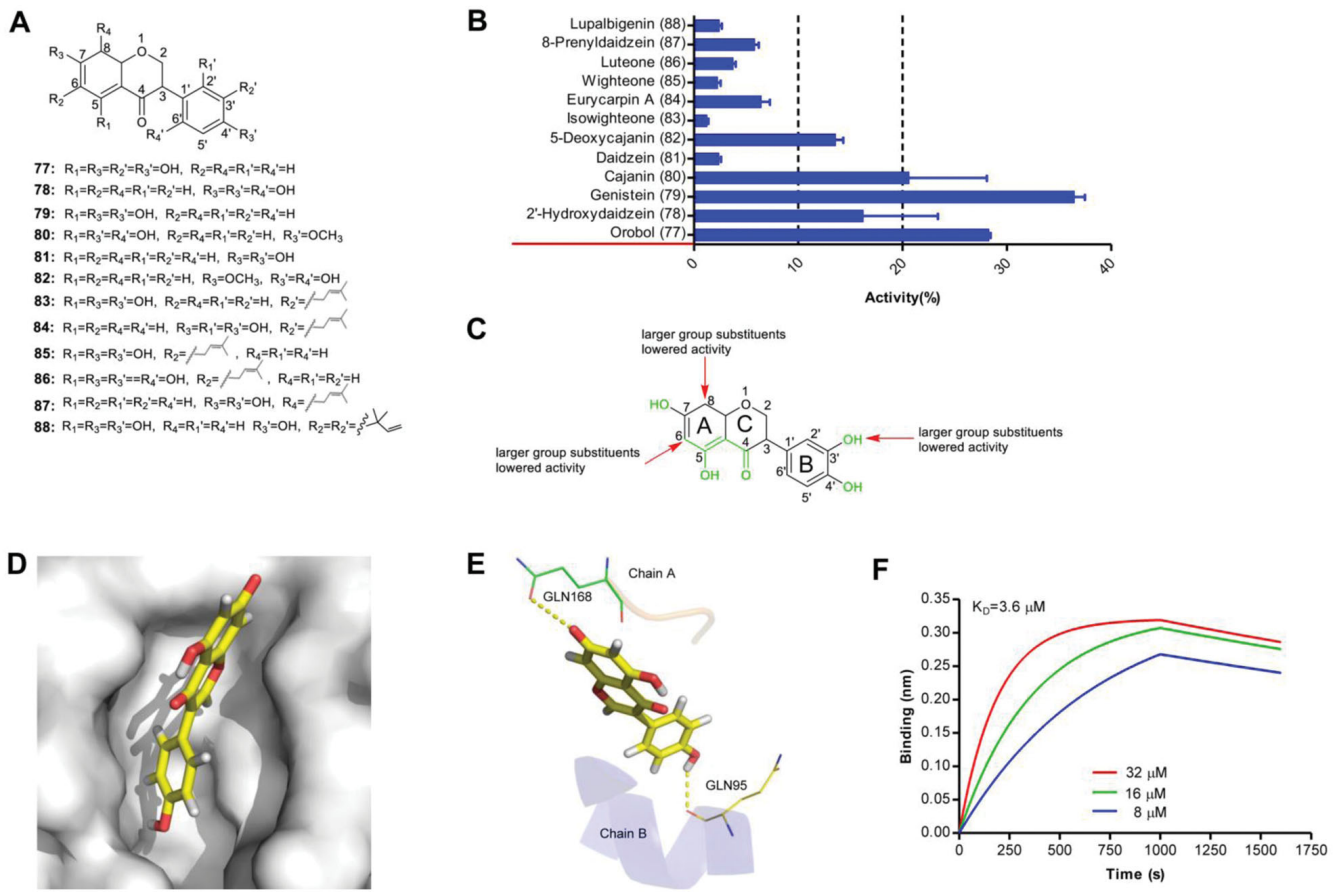
(A) Chemical structures of the studied isoflavones. (B) The inhibitory effects of 12 isoflavones (50 μM, final concentration) against IN-LEDGF/p75 interaction. (C) Proposed semi-quantitative structure-activity relationships for isoflavones. Structural features decreasing the inhibition of IN-LEDGF/p75 interaction are marked in red colour. Structural characteristics increasing the inhibitory potential of isoflavones are shown in green. Predicted binding modes of genistein *via* Chimaera. The binding pocket of the IN CCD was illustrated as (D) surface and (E) thin lines for key residues (PDB accession code 4NYF). Genistein is coloured yellow. Yellow dashed lines represent H-bonding interactions between the genistein and receptor. (F) Sensorgram of the interaction between the immobilised IN and genistein.

Effects of 12 isoflavones on IN–LEDGF/p75 interaction were tested in an HTRF-based assay (Figure 6A). Among these investigated compounds, only 3 compounds showed pronounced (>20%) inhibitory activity (Figure 6B). The catechol group played an important role in the blockage of protein-protein interaction (77 vs 78, 79, 80, 81). The larger substituents at the positions 6, 8, 3′-site led to the drop in protein-protein interaction disruption. The structural features influencing the flavans′ inhibitory activities on IN-LEDGF/p75 interaction was shown in Figure 6C. The predicted binding modes of one representative compound genistein were shown in Figure 6D and E. Based on molecular docking analysis, we observed that the 7-hydroxyl group of the A-ring formed hydrogen bonds with the main-chain amides of GLU168 in chain A, and a 4′-hydroxyl group of B-ring formed a hydrogen bond with residue GLN95 in the chain B. BLI analysis confirmed the direct binding between genistein and IN with a K_D_ value of 3.6 µM (Figure 5F).

### 3.7. IN-LEDGF/p75 interaction inhibitory activity of chalcones

**Figure 7.**
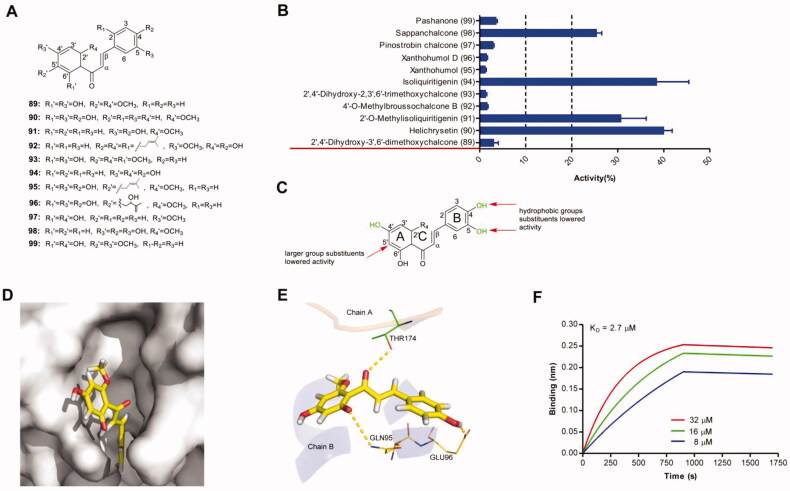
(A) Chemical structures of the studied chalcones. (B) The inhibitory effects of 12 isoflavones (50 μM, final concentration) against IN-LEDGF/p75 interaction. (C) Proposed semi-quantitative structure-activity relationships for chalcones. Structural features decreasing the inhibition of IN-LEDGF/p75 interaction are marked in red colour. Structural characteristics increasing the inhibitory potential of chalcones are shown in green. Plausible binding modes of helichrysetin *via* Chimaera. The binding pocket of the IN CCD was illustrated as (D) surface and (E) thin lines for key residues (PDB accession code 4NYF). Helichrysetin is coloured yellow. Yellow dashed lines represent H-bonding interactions between the helichrysetin and receptor. (F) Sensorgram of the interaction between the immobilised IN and helichrysetin.

Eleven chalcones (Figure 7A) were tested for their ability to disrupt IN–LEDGF/p75 interaction. Among these evaluated compounds, 4 compounds showed more potent activity against IN–LEDGF/p75 interaction (Figure 7B). The larger substituents at the position 5′-site led to the drop in protein-protein interaction disruption (e.g. compounds 89, 92, 93, 95, 96, 99). The catechol structure or 4′-hydroxy was beneficial for the protein-protein interaction disruption (e.g. compounds 90, 91, 94, 98). These structural features influencing the flavans′ inhibitory activities on IN-LEDGF/p75 interaction was shown in Figure 7C. As shown in the predicted binding modes of one representative compound helichrysetin (Figure 7D and E), it could be observed that the carbonyl group of the C-ring formed hydrogen bonds with the main-chain amides of THR174 in chain A, and 6′-hydroxyl group of A-ring formed a hydrogen bond with residue GLN95 and a 4-hydroxyl group of B-ring formed a hydrogen bond with residue GLU96 in the chain B. BLI assay confirmed that genistein can bind directly to IN with a K_D_ value of 2.7 µM (Figure 7F).

## 4. Discussion

The LEDGF/p75 binding pocket of HIV-1 IN was recently investigated for the development of a new class of IN inhibitors. Several small molecules have been identified as inhibitors of the IN-LEDGF/p75 interaction, including KF116, MUT-A, BI224436, CX14442, BI-D, LEDGIN 1, et al. [26,27]. Flavonoids, as plant metabolites, have been shown to block the enzymatic activity of IN and IN-LEDGF/p75 interaction [16,28]. The present research compared the effects of 99 compounds belonging to different flavonoidic subclasses on IN-LEDGF/p75 interaction to establish an accurate structure-activity relationship.

Different structural classes of selected flavonoids showed different inhibitory activity against IN-LEDGF/p75 interaction. Among them, flavonol and flavone performed best, while isoflavones exhibited the weakest activity. Although these compounds have different structures, their inhibition of the interaction shared the common features: the presence of hydroxyl group in C3, C4, C5 and C7 in the A-ring, C4′ and C5′ in the B-ring, the carbonyl group in the C-ring, contributed to the inhibitory activity of flavonoids on IN-LEDGF/p75 interaction, whereas hydrophobic groups substituents at C6 and C7 in the A-ring, C4′ and C5′ in the B-ring reduced the inhibitory activity of flavonoids on IN-LEDGF/p75 interaction. Therefore, it may be inferred from the above results that disruption of IN-LEDGF/p75 interaction by flavonoids is strongly related to the position and nature of the substituents (summarized in Table 1), which is consistent with the previous study [16].

**Table 1.** Summary of the important residues of IN and the important substitution in the different compounds leading to the best compound binding.

Flavonoidic subclass	Key residues for binding		Structure features affecting ILI^a^	
	Chain A	Chain B	↑	↓
Flavonol	HIS171, THR174	GLN 95	7-OH, CG^b^ & KG^c^, KG & PG^d^	LS^e^ in 7-OH; HGS^f^ in 4′, 5′-OH
Flavones	GLU170	THR125	7-OH, CG & KG	MGS^g^ in 6-OH; HGS in 7, 4′, 5′-OH
Flavanonols	GLU170	ALA128, GLN95	3, 7-OH, KG & PG	MGS in 3, 6-OH; HGS in 4′, 5′-OH
Flavanones	GLU170, HIS171		7-OH, CG, KG	LS in position 6, 8; MGS in 7-OH
Flavans	GLU170, GLN168	GLN95	7-OH, CG, PG	HGS in 4′, 5′-OH
Isoflavones	GLN168	GLN95	7-OH, CG, KG	LS in position 6, 8, 3′-OH
Chalcones	THR174	GLN95, GLU96	4′-OH, CG	LS in position 5′, HGS in 4, 5-OH

**^a^**ILI, IN-LEDGF/p75 interaction;↑, increase, ↓, decrease. ^b^CG, catechol group. ^c^KG, ketoenol group. ^d^PG, pyrogallol group. ^e^LS, larger substituent. ^f^HGS, hydrophobic groups substituent. ^g^MGS, methoxyl group substituent.

Generally, inhibitors of IN-LEDGF/p75 interaction bind to the LEDGF/p75 binding pocket in IN CCD dimer interface. BLI assay confirmed that six representative flavonoids are directly bound to IN. Molecular docking analysis revealed that the representative flavonoids excluding genistein bound to hot spots of IN, including residues GLU171, HIS171, THR174 and ALA128 (summarized in Table 1), as most inhibitors of IN-LEDGF/p75 interaction did [25]. While, genistein is bound to GLN168 and GLN95 rather than any hot spots of IN, which may lead to its worst inhibitory activity against IN-LEDGF/p75 interaction. Moreover, luteolin is also bound to THR125 which is located on the edge of the LEDGF binding pocket. A previous study indicated that reside THR125 of IN plays an important role in the activity of inhibitors against IN-LEDGF/p75 interaction by interacting with the part opposite to the carboxylic acid side chain of these compounds [29]. We speculated that disruption of IN-LEDGF/p75 interaction by luteolin was partially related to its interaction with THR125.

To the best of our knowledge, our study is the first comprehensive investigation of the effects of flavonoids on IN-LEDGF/p75 interaction. The scope of flavonoids used in this research was broad, including chemical classes such as flavonols, flavanones, flavanonols, flavones, flavan, chalcones and isoflavones. However, two classes of flavonoids, including homoisoflavone and xanthones, were not studied in the present study, which should be investigated in our future research.

## 5. Conclusion

Our study, for the first time, quantitatively compared the effect of flavonoids belonging to seven flavonoidic subclasses on IN-LEDGF/p75 interaction. SAR analysis suggested that functional groups were important for the inhibitory activity of flavonoids against IN-LEDGF/p75 interaction. These results strongly suggested that flavonoids are a valuable source of inhibitors of IN-LEDGF/p75 interaction and are worthy of further investigation.

## Supplementary Material

Supplemental Material

## Data Availability

The data that support the findings of this study are openly available in [Mendeley data] at [https://data.mendeley.com/datasets/gppgrc8hwg/draft?a=da1ea5a8-4886-4893-a2a2-9fe10d496404].
