# Supplementary material for: Evaluation of a flavonoid library for inhibition of interaction of HIV-1 integrase with human LEDGF/p75 towards a structure–activity relationship
Source: Ann Med. 2022 Jun 6;54(1):1590–600. doi: 10.1080/07853890.2022.2081869 (PMC9176681; doi:10.1080/07853890.2022.2081869)
Supplement: Supplemental Material [file IANN_A_2081869_SM4779.docx]

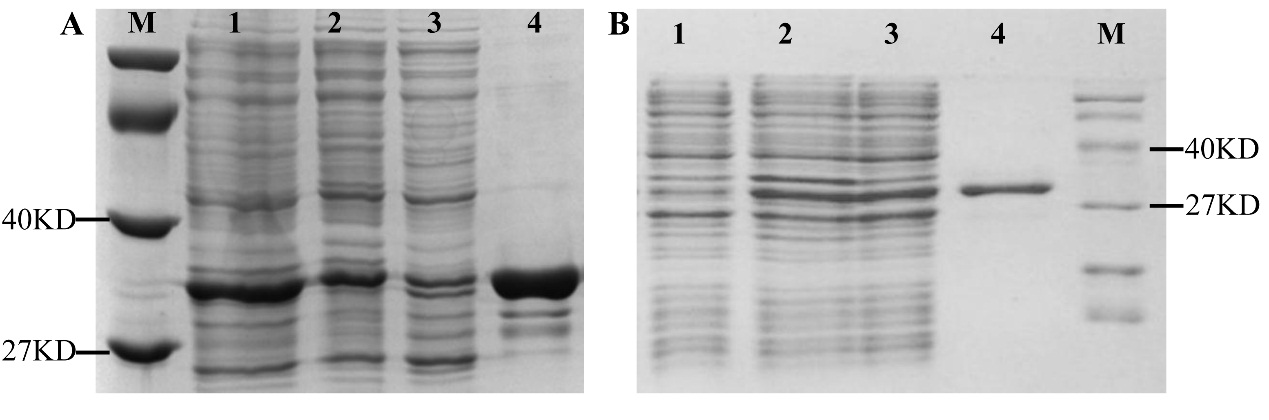
Figure S1 Figure S1 12% SDS-PAGE analysis of purified GST-IBD (A) and His_6_-IN (B) . Purified protein was shown in lane 4 on each gel.

Figure S2


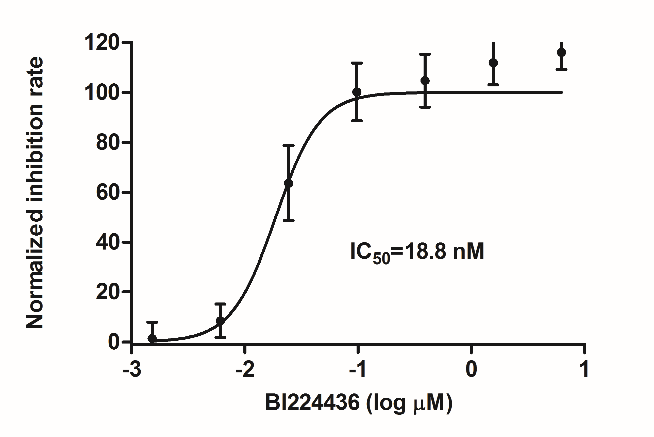


Figure S2 Dose-response curves of BI224436 on IN-LEDGF/p75 interaction. Error bars represent SD from 3 replicate values.
